# Defining the determinants of endurance running performance in the heat

**DOI:** 10.1080/23328940.2017.1333189

**Published:** 2017-05-25

**Authors:** Carl A. James, Mark Hayes, Ashley G. B. Willmott, Oliver R. Gibson, Andreas D. Flouris, Zachary J. Schlader, Neil S. Maxwell

**Affiliations:** a Environmental Extremes Laboratory, Centre for Sport and Exercise Science and Medicine (SESAME), University of Brighton, Eastbourne, UK; b National Sports Institute of Malaysia (Institut Sukan Negara), Bukit Jalil Stadium, Kuala Lumpur, Malaysia; c Centre for Human Performance, Exercise and Rehabilitation (CHPER), Brunel University London, London, UK; d FAME Laboratory, Department of Exercise Science, University of Thessaly, Trikala, Greece; e Centre for Research and Education in Special Environments, Department of Exercise and Nutrition Sciences, University at Buffalo, Buffalo, NY, USA

**Keywords:** endurance, heat stress, lactate threshold, performance, running, thermoregulation, V̇O_2max_

## Abstract

In cool conditions, physiologic markers accurately predict endurance performance, but it is unclear whether thermal strain and perceived thermal strain modify the strength of these relationships. This study examined the relationships between traditional determinants of endurance performance and time to complete a 5-km time trial in the heat. Seventeen club runners completed graded exercise tests (GXT) in hot (GXTHOT; 32°C, 60% RH, 27.2°C WBGT) and cool conditions (GXTCOOL; 13°C, 50% RH, 9.3°C WBGT) to determine maximal oxygen uptake (V̇O_2max_), running economy (RE), velocity at V̇O_2max_ (vV̇O_2max_), and running speeds corresponding to the lactate threshold (LT, 2 mmol.l^−1^) and lactate turnpoint (LTP, 4 mmol.l^−1^). Simultaneous multiple linear regression was used to predict 5 km time, using these determinants, indicating neither GXTHOT (R^2^ = 0.72) nor GXTCOOL (R^2^ = 0.86) predicted performance in the heat as strongly has previously been reported in cool conditions. vV̇O_2max_ was the strongest individual predictor of performance, both when assessed in GXT_HOT_ (*r* = −0.83) and GXT_COOL_ (*r* = −0.90). The GXTs revealed the following correlations for individual predictors in GXT_HOT_; V̇O_2max_ *r* = −0.7, RE *r* = 0.36, LT *r* = −0.77, LTP *r* = −0.78 and in GXT_COOL_; V̇O_2max_ *r* = −0.67, RE *r* = 0.62, LT *r* = −0.79, LTP *r* = −0.8. These data indicate (i) GXT_HOT_ does not predict 5 km running performance in the heat as strongly as a GXT_COOL_, (ii) as in cool conditions, vV̇O_2max_ may best predict running performance in the heat.

## Introduction

An individual's free-paced running velocity represents an observable behavioral consequence of collective physiologic, psychological, and tactical feedback. Under normothermic conditions, this behavioral response is highly associated with physiologic markers of endurance performance, such as lactate threshold (LT), lactate turnpoint (LTP), running economy (RE), and maximum oxygen uptake (V̇O_2max_).^1-9^ Thus, these factors have been termed as the determinants of endurance performance.^1,7,8,10,11^

The determinants of the endurance performance model^1^ demonstrate how an individual's V̇O_2max_ determines the upper limit of aerobic metabolism, beneath which the LTP corresponds to the fractional utilization of V̇O_2max_ (%V̇O_2max_) that can be sustained. Running velocity is then determined by how efficiently the corresponding oxidative adenosine triphosphate turnover at the fractional utilization of V̇O_2max_ is converted to locomotion (i.e., RE). During an outdoor 16 km race, McLaughlin et al.^8^ reported that these determinants collectively accounted for 95.4% of the variation in performance. The composite measure of RE and velocity at V̇O_2max_, (vV̇O_2max_) explained 94.4% of the variation in performance time, while V̇O_2max_ and LT each accounted for ∼90%.^8^

Endurance running in hot and humid environmental conditions is characterized by elevated thermoregulatory, cardiovascular, and perceived exertional strain, which typically leads to a performance decrement, relative to cooler conditions.^12^ Whether environmental heat stress and the consequential elevated physiologic strain modifies the relationship between the determinants of endurance performance and free-paced running velocity is unclear. There is some evidence indicating that the sustainable exercise intensity in the heat for 1 hour may be accurately predicted by the LTP,^13^ while the progressive decrement in V̇O_2max_ also appears to be important in determining absolute exercise intensity (i.e., running velocity, power output), with Périard & Racinais^14^ suggesting individuals maintain a similar relative exercise intensity (%V̇O_2max_) in the heat, as in cool conditions. Therefore, individual determinants of endurance performance, such as the LTP and V̇O_2max_, appear to remain important for predicting endurance performance in the heat. However, specific thermal factors such as perceived thermal discomfort have also been proposed as determinants of self-paced exercise in the heat.^15^ Behavioral thermoregulation is a volitional process that typically presents through reducing metabolic heat production (i.e., reducing exercise intensity) and, thus, body heat storage during endurance exercise in response to unfavorable thermal discomfort.^16^ Therefore, behavioral thermoregulation may confound existing relationships between physiologic markers and endurance performance when exercising in the heat.^17^ Such a suggestion is in accordance with indices of perceived strain, such as thermal sensation and discomfort, being determined somewhat independently of traditional physiologic markers, for example, in advance of changes in core body temperature.^18^ Accordingly, highlighting an integrated behavioral thermoregulatory response, Flouris and Schlader^17^ suggested that elevated skin temperature (T_SKIN_) elicits concomitant effects on both perceived strain (i.e., thermal discomfort and/or exertion (RPE))^16^ and cardiovascular strain,^19,20^ indicating that performance in the heat is influenced by both behavioral consequences and systemic physiologic limitations.

Therein, a heightened perceived strain may reduce the variation attributable to the traditional physiologic determinants, such that the specific determinants of endurance performance in the heat are not clearly defined. Such information would facilitate a more informed application of thermal interventions, such as precooling and heat acclimation, for alleviating physiologic versus perceived strain, but also aid performance prediction and training prioritization. Therefore, this study investigated the relationship between V̇O_2max_, LT, LTP, RE, vV̇O_2max_, and 5-km treadmill time trial (TT) performance, when the determinants are measured in both hot (32°C, 60%) and cool (13°C, 50%) conditions. The ultimate goal of this work is to inform on the appropriateness of using the traditional determinants of endurance performance model for research on endurance running in the heat.

## Methods

This study on predicting 5-km running performance from the determinants of endurance performance is part of a recently published larger study of heat acclimation and performance.^21^ However, the current study investigates different hypotheses, focused on predicting performance in the heat, when the determinants have been measured in hot and cool conditions.

### Participants

Seventeen, amateur, club runners (16 male, 1 female), who trained a minimum of three times per week for the previous 2 months, volunteered for this study (mean ± SD: age 32 ± 13 years, stature 177 ± 6 cm, mass 71.9 ± 8.9 kg, body fat percentage 11.2 ± 2.1%, V̇O_2max_ 61.0 ± 6.2 mL.kg^−1^.min^−1^, recent 5 km time 20:25 ± 1:42 min). Testing occurred in the UK spring, therefore participants were not naturally heat acclimated and were entering the competition season. The female participant completed all trials during the follicular phase of the menstrual cycle, verified via a questionnaire. Each participant provided written-informed consent and institutional ethical approval was issued in accordance with the Declaration of Helsinki (2013). Participants avoided intense exercise, alcohol and caffeine for 48 hours before testing and arrived hydrated, verified through urine analysis using a refractometer (< 1.020, specific gravity refractometer 32, Atago, USA) in accordance with Sawka et al.^22^

### Experimental design

Participants visited the laboratory on five occasions, comprising two familiarization visits and three experimental trials. On the first visit, a four-site skin fold caliper assessment was completed across the iliac crest, subscapular, triceps, and biceps, for the estimation of body fat percentage in accordance with the calculation of Siri.^23^ Participants were then familiarized with a graded exercise test in hot and humid conditions (GXT_HOT_; 32°C, 60% relative humidity (RH), 27.2°C wet bulb globe temperature (WBGT)). During the second familiarization visit, participants completed a 5-km treadmill TT under the same environmental conditions. Experimental trials began 2 weeks after familiarizations to control against thermal adaptations from repeated visits^24^ and occurred at a similar time of the day for each participant to control for diurnal core temperature fluctuation.^25^

During the first experimental trial, participants completed a GXT in cool conditions (GXT_COOL_; 13°C, 50% RH, 9.3°C WBGT) to assess the determinants of endurance performance (LT, LTP, RE, V̇O_2max_, vV̇O_2max_). At least 2 d later, participants completed GXT_HOT_, the same test in hot and humid conditions (32°C, 60% RH, 27.2°C WBGT). The final trial occurred 3–4 d later, where participants completed a 5-km treadmill TT in the same hot conditions. All trials were conducted in the same order and within an environmental chamber (WatFlow control system TISS, Hampshire, UK).

### Graded exercise test

**Figure 1.**
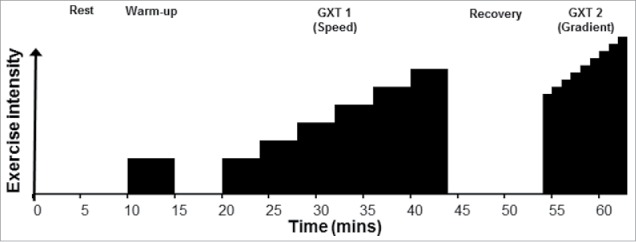
Time course of graded exercise test. The entire protocol took place within the hot or cool environment.

A graded exercise test, split into two parts, GXT 1 and GXT 2, was adopted, as described previously by Jones.^26^ Following a 10-min rest in the hot or cool environment and a 5-min low-intensity warm-up (matched across both trials), GXT 1 was a submaximal incremental speed protocol, followed by GXT 2; an incremental gradient protocol to volitional exhaustion (Fig. 1). Starting speed during GXT 1 was determined from recent running performance and the familiarizations, to ensure an initial steady-state blood lactate response, while achieving an exponential increase in blood lactate concentration (> 4 mmol.l^−1^) within 6–8 exercise stages. Each stage involved 3 min of exercise, followed by 1-min rest, for blood sampling. Stages were separated by increments of 1 km.h^−1^. All tests occurred on a motorized treadmill (Woodway ELG2, Weil am Rhein, Germany) set at 1% incline, to replicate outdoor running.^27^ Following a 10-min rest in the hot or cool environment, GXT 2 began 2 km.h^−1^ below the previous final speed, with gradient increasing by 1% each min. Participants were not permitted to drink and were blinded to all forms of feedback.

### 5-km time trial

Upon arrival, participants rested in the hot environment for 10 min, before completing a 5 min, self-selected warm-up. Standardized instructions were given at the start of the trial and nothing thereafter, with participants instructed to “give your all,” “pace yourself throughout the trial,” and “adjust speed as you see fit” as per similar research.^28^ Participants straddled the treadmill belt, increased to the individual's average speed from the familiarization, to maintain a consistent blinded starting speed. The trial began when the participant began running, with speed adjustment immediately permitted *ad libitum* (increment 0.2 km.h^−1^) and total distance continuously displayed. The treadmill gradient was fixed at 1%. Participants were given no other feedback and did not drink during trials. Pilot testing within our laboratory, using a similar cohort, indicated a typical error for this trial of 16 s (1.2%).

### Physiological and perceptual measures

During all trials, sweat loss was estimated from pre- and post-exercise nude body mass to the nearest 0.01 kg using precision scales (GFK 150, Adam Equipment, Milton Keynes, UK). Core temperature (T_CORE_) was measured using disposable rectal probes (Henleys Medical, UK), inserted to 10 cm beyond the anal sphincter and connected to a data logger (Model 401, Yellow Springs Instruments, Ohio, USA). Telemetry thermistors (U-type connected to Gen II GD38 transmitter, Eltek, UK) were attached to the pectoralis major, biceps brachii, rectus femoris, and gastrocnemius for measuring T_SKIN_ to calculate a weighted mean skin temperature.^29^ Heart rate (HR) was monitored continuously using a Polar 810i heart rate monitor (Kempele, Finland). HR, T_CORE_, T_SKIN_, rating of perceived exertion (RPE^30^), and thermal sensation (TS, 0 = unbearably cold to 8 = unbearably hot^31^) were noted at the end of each stage during GXT 1 and every km during the TT.

During the GXTs, running speeds at 2 and 4 mmol.l^−1^ were calculated by solving the polynomial regression equation for blood lactate concentration versus speed at 2 and 4 mmol.l^−1^, denoting the lactate threshold (LT) and lactate turnpoint (LTP), respectively.^32^ This approach accounted for differences in the number of stages completed, removed subjectivity of experimenter identification and provided precision to less than 1 km.h^−1^. Fingertip blood samples at the end of each stage were analyzed immediately (2300 analyzer, Yellow Springs Instruments, Ohio, USA). Ventilatory gases were measured using 30 s fixed-time averages (Metalyzer 3B, Cortex, Leipzig, Germany), with the two values from the final min of each stage used for RE. Average RE (mL O_2_.kg^−1^.km^−1^) was calculated across the first five exercise stages, using the two fixed 30 s averages from the final minute of each stage. During GXT 2, the highest 30 s moving average represented V̇O_2max_. A V̇O_2max_, not V̇O_2peak_, was accepted when a V̇O_2_ plateau (< 2 mL.kg^−1^.min^−1^ across two successive 30 s fixed-time averages was observed).^32^ While a subsequent verification phase is recommended for the robust assessment of V̇O_2max_,^33^ the precise consequences of heat strain cannot be accurately replicated, which would be necessary given the strong relationship between heat strain and V̇O_2max_ decrement.^12^ Therefore, in the absence of a plateau, a test was deemed maximal if three out of the following four criteria were met; blood lactate concentration >8 mmol.l^−1^, HR within 10 beats of age-predicted maximum, respiratory exchange ratio >1.1, and RPE at or above 19, as we have previously adopted.^21,34^ Velocity at V̇O_2max_ was calculated by multiplying V̇O_2max_ (mL.kg^−1^.min^−1^) by 60 and dividing by the average RE.^26^

### Data analyses

All outcome variables were assessed for normality and sphericity before further analysis. Data were analyzed using SPSS (version 21, SPSS Inc., Illinois, USA) with statistical significance set at *p*<0.05 and presented as means ( ± SD). Initially, paired Student's *t*-tests were used to indicate differences in the number of V̇O_2max_ and V̇O_2peak_ tests, between conditions. Pearson's correlations (r) were used to examine for statistically significant relationships between the individual determinants of endurance performance and 5-km time. Following determination of a significant linear relationship, statistically significant variables were entered into a simultaneous multiple linear regression, with LT, LTP, RE, V̇O_2max_ and vV̇O_2max_ as the predictor variables and 5-km time as the dependent variable. The magnitude of difference between related, single samples is demonstrated by Cohen's *d_av_*, in accordance with the recommendations of Lakens.^35^ Correlations greater than 0.5 are considered *large*, 0.5–0.3 *moderate* and 0.3–0.1 *small*, in accordance with Cohen.^36^

## Results

Technical faults resulted in no data for one measure of both V̇O_2max_ and RE (different individuals). Consequently, correlations are derived from *n* = 17 for LT & LTP, *n* = 16 for V̇O_2max_ & RE, and *n* = 15 for vV̇O_2max_. All predictor variables from both GXT_HOT_, GXT_COOL_, and 5-km TT performance were normally distributed. Based on the predefined criteria, no difference (*p* = 0.08) was observed in the prevalence of V̇O_2max_ and V̇O_2peak_ tests between GXT_COOL_ (V̇O_2max_ = 15, V̇O_2peak_ = 2) and GXT_HOT_ (V̇O_2max_ = 12, V̇O_2peak_ = 5).

### GXT physiologic responses

**Figure 2.**
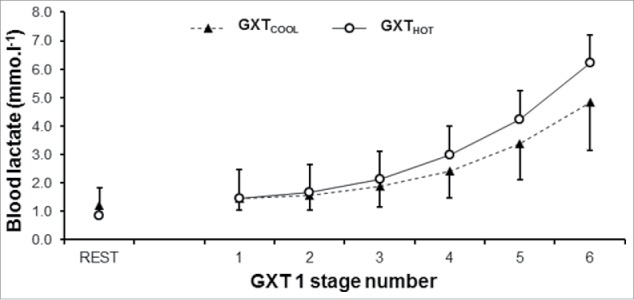
Mean ( ± SD) blood lactate response during graded exercise in hot conditions, compared with cool conditions. Error bars represent one standard deviation.

**Figure 3.**
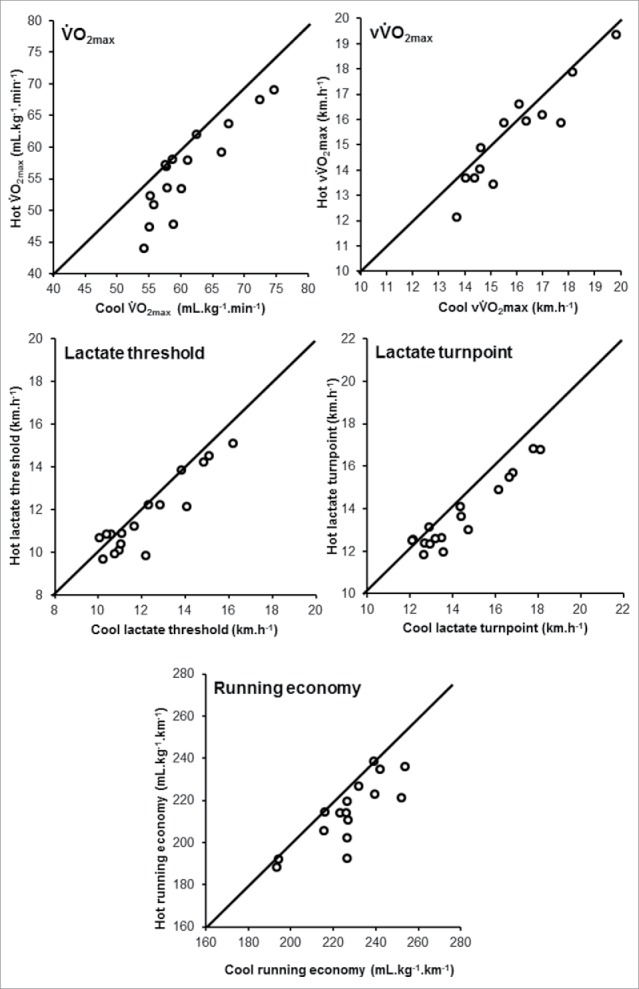
Individual plots demonstrating decrements from heat stress for determinant of endurance performance.

Incremental running in GXT_HOT_ elicited a holistically elevated physiologic strain compared with GXT_COOL_, as shown in Table 1. For complete physiologic results, please see our recently published paper.^21^ Briefly, blood lactate concentration was elevated in GXT_HOT_ (Fig. 2), resulting in a −0.6 (0.8) km.h^−1^ reduction in the LT and −0.7 (0.7) km.h^−1^ the LTP. V̇O_2max_ was also impaired in GXTHOT (−4.6 ± 3.3 mL.kg^−1^.min^−1^), as was vV̇O2max (−0.6 ± 0.7 km.h^−1^). RE improved in GXTHOT, with the mean metabolic cost of running reduced by 12.3 ± 10.1 mL.kg^−1^.km^−1^, in the heat. Individual data demonstrating the impairments arising from heat stress to each of the determinants of 5-km performance are shown in Fig. 3.

**Table 1.** Mean (± SD) physiologic and perceptual responses when measured in hot (GXT_HOT_) and cool conditions (GXT_COOL_). * represents statistical difference (*p* <0.05).

	GXT_COOL_ (13°C)	GXT_HOT_ (32°C)	*d_av_*
GXT 1			
Lactate threshold speed (2 mmol.l^−1^) (km.h^−1^)	12.3 (1.9)	11.7 (1.8)*	0.31
Lactate turnpoint speed (4 mmol.l^−1^) (km.h^−1^)	14.4 (2.0)	13.7 (1.7)*	0.40
Running economy (mL.kg^−1^.km^−1^)	227 (17)	215 (16)*	0.75
T_SKIN_ (°C)	28.3 (1.4)	35.3 (1.1)*	5.69
Δ T_CORE_ (°C)	1.25 (0.41)	1.67 (0.39)*	1.03
Core:skin gradient (°C)	10.3 (1.1)	2.6 (0.8)*	8.40
RER	1.04 (0.07)	1.12 (0.13)	0.73
Thermal sensation	5.4 (1.0)	6.9 (0.8)*	1.60
RPE	15.9 (1.3)	17.7 (1.4)*	1.36
GXT 2			
V̇O_2max_ (mL.kg^−1^.min^−1^)	61.0 (6.2)	56.3 (7.1)*	0.70
vV̇O_2max_ (km.h^−1^)	16.1 (2.1)	15.8 (2.3)*	0.23
Finishing T_CORE_ (°C)	38.53 (0.39)	38.88 (0.27)*	1.05
Finishing HR (b.min^−1^)	186 (12)	189 (9)*	0.27

### 5-km time trial in the heat

Physiological and perceptual responses from the 5-km TT in hot conditions are shown in Table 2.

**Table 2.** Mean ( ± SD) physiologic and perceptual responses during 5-km time trial in hot conditions.

	Rest	1 km	2 km	3 km	4 km	5 km
HR (b.min^−1^)	55 (8)	165 (12)	172 (11)	176 (9)	177 (8)	186 (9)
% HRmax	29 (4)	88 (4)	92 (4)	94 (3)	95 (5)	99 (5)
T_CORE_ (°C)	37.1 (0.2)	37.6 (0.2)	38.1 (0.2)	38.6 (0.3)	39.0 (0.3)	39.3 (0.3)
T_SKIN_ (°C)	33.4 (0.9)	34.9 (1.1)	35.1 (1.3)	35.3 (1.3)	35.5 (1.3)	35.7 (1.2)
Core:skin gradient (°C)	3.7 (0.9)	2.5 (0.7)	2.6 (1.0)	2.9 (1.1)	3.2 (1.0)	3.4 (1.0)
Thermal sensation	4.3 (0.4)	5.6 (0.7)	6.3 (0.8)	6.8 (0.9)	7.0 (0.8)	7.4 (0.6)
RPE	—	14.1 (1.4)	15.3 (1.6)	16.1 (1.4)	17.3 (1.1)	18.5 (1.4)
Sweat rate (l.hr^−1^)	—	—	—	—	—	2.2 (0.8)

### Predictions from GXT_HOT_

**Figure 4.**
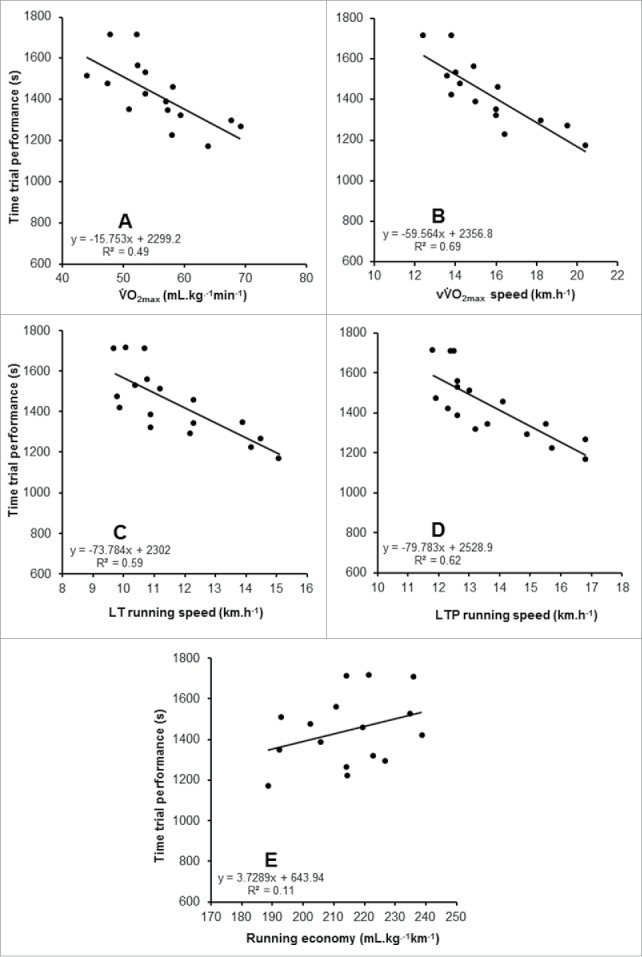
Relationships between the determinants of endurance performance when measured in a hot environment (GXT_HOT_) and 5-km time trial performance in the heat. A – V̇O_2max_, B – vV̇O_2max_, C – lactate threshold (LT), D – lactate turnpoint (LTP), E – running economy.

When measured in GXT_HOT_, LT, LTP, V̇O_2max_, and vV̇O_2max_ were identified as statistically significant predictors of TT performance, but RE was not. The strongest individual predictor from GXT_HOT_ was vV̇O_2max_ (*r* = −0.83). These data are shown in Table 3 and Fig. 4. Due to the absence of a statistically significant relationship between RE and TT performance, all variables aside of RE, were entered into a simultaneous multiple linear regression, revealing a significant relationship with TT performance (F_[4, 10]_ = 6.508, *p* = 0.008, R^2^ = 0.72, standard error of the estimate (SEE) = 106 s). The model revealed the following formula for predicting 5-km performance based on measures derived from GXT_HOT_.

$$5\;\text{km}\;\text{time}\left(\text{s}\right)=2352.608-(2.377\;\dot{\text{V}}{\text{O}}_{2\max})-(48.629\;\text{v}\dot{\text{V}}{\text{O}}_{2\max})-(69.266\;\text{LT})+(57.706\;\text{LTP}).$$

**Table 3.** The determinants of endurance performance (mean ± SD), with respective correlations (*r*) and statistical significance (*p*) for predicting running performance in existing literature using trained runners and from this study (shown in bold). Where fixed blood lactate concentrations other than 2 and 4 mmol.l^−1^ have been used, the respective value is stated.

Study	n	Cohort characteristics	Distance (km)	V̇O_2max_ (mL.kg^−1^.min^−1^)	vV̇O_2max_ (km.h^−1^)	LT (km.h^−1^)	LTP (km.h^−1^)	RE (mL.kg^−1^.km^−1^)
This study (GXT_HOT_)	**17**	**Heterogeneous**	**5**	**56.3 (7.1)**	**15.8 (2.3)**	**11.7 (1.8)**	**13.7 (1.7)**	**215 (16)**
		**Trained**		***r* = −0.70**	***r* = −0.83**	***r* = −0.77**	***r* = −0.78**	***r* = 0.36**
		**(5 km <22 min)**		***p* = 0.003**	***p* < 0.001**	***p* < 0.001**	***p* < 0.001**	***p* = 0.205**
This study (GXT_COOL_)	**17**	**Heterogeneous**	**5**	**61.0 (6.2)**	**16.1 (2.1)**	**12.3 (1.9)**	**14.4 (2.0)**	**227 (17)**
		**Trained**		***r* = −0.67**	***r* = −0.90**	***r* = −0.79**	***r* = −0.80**	***r* = 0.62**
		**(5 km <22 min)**		***p* = 0.004**	***p* < 0.001**	***p* < 0.001**	***p* < 0.001**	***p* = 0.011**
Morgan et al. (1986)^70^	13	Heterogeneous	10	66.2 (*N/A*)	*N/A*	*N/A*	*N/A*	*N/A*
		Well trained		*r* = −0.55	*r* = −0.78		*r* = −0.85	*r* = 0.30
		(10 km = ∼31 ± 2 min)		*p* = <0.05	*p* = 0.002		*p* = −0.002	*p* = 0.29
Morgan et al. (1989)^37^	10	Homogenous		64.8 (2.1)	*N/A*	*N/A*	*N/A*	*N/A*
		Well trained		*r* = −0.45	*r* = −0.87		*r* = −0.82	*r* = 0.64
		(10 km = ∼32 ± 1.5 min)		*p* = >0.05	*p* = <0.01		*p* = <0.01	*p* = <0.05
Noakes et al. (1990)^38^	43	Heterogeneous	10	66.2 (8.0)	21.3 (2.0) (PTV)	*N/A*	16.0 (2.4) (INFL)	192 (12)
		Well trained		*r* = −0.55	*r* = −0.94		*r* = −0.91	*r* = 0.41
		(10 km = 35 ± 4 min))		*p* = <0.01	*p* = <0.01		*p* = <0.01	*p* = <0.01
Yoshida et al. (1993)^49^	57	Heterogeneous	3	58.7 (3.8)	17.2 (0.9)	13.6 (0.9) (INFL)	16.0 (0.8)	197 (12)
		Well trained		*r* = −0.51	*r* = −0.75	*r* = −0.77	*r* = −0.60	R = 0.24
		(3 km = ∼10.2 min)		*p* = <0.01	*p* = <0.01	*p* = <0.01	*p* = <0.01	*p* = >0.05
Grant et al. (1997)^43^	16	Heterogeneous		73.3 (6.7)	20.7 (2.1)	16.0 (1.8) (INFL)	17.1 (1.9)	*N/A*
		Well trained		*r* = −0.70	*r* = −0.86	*r* = −0.93	*r* = −0.93	*r* = 0.53
		(3 km <10.5 min)		*p* = <0.05	*p* = <0.05	*p* = <0.05	*p* = <0.05	*p* = <0.05
Jones & Doust (1998)^6^	13	Homogeneous	8	60.7 (4.0)	18.1 (0.4)	15.1 (0.3) (INFL)	16.1 (0.2)	*N/A*
		Well trained		*r* = −0.69	*r* = −0.93	*r* = −0.93	*r* = −0.81	
		(8 km ∼30 min)		*N/A*	*N/A*	*N/A*	*N/A*	
McLaughlin et al. (2010)^8^	17	Heterogeneous	16	56.6 (7.2)	16.6 (2.8)	12.3 (2.2)	14.8 (2.2) 3 mmol.l^−1^	204 (12)
		Trained		*r* = −0.91	*r* = −0.97	*r* = −0.85	*r* = −0.89	*r* = 0.81
		(16 km = ∼1 h 08 min)		*p* = *N/A*	*p* = *N/A*	*p* = *N/A*	*p* = *N/A*	*p* = *N/A*

N.B. “INFL” represents determination of the LT or LTP based on an inflection point, where blood lactate concentration exceeds steady state (LT) or demonstrates an exponential increase (LTP). N/A = data not stated.

### Predictions from GXT_COOL_

**Figure 5.**
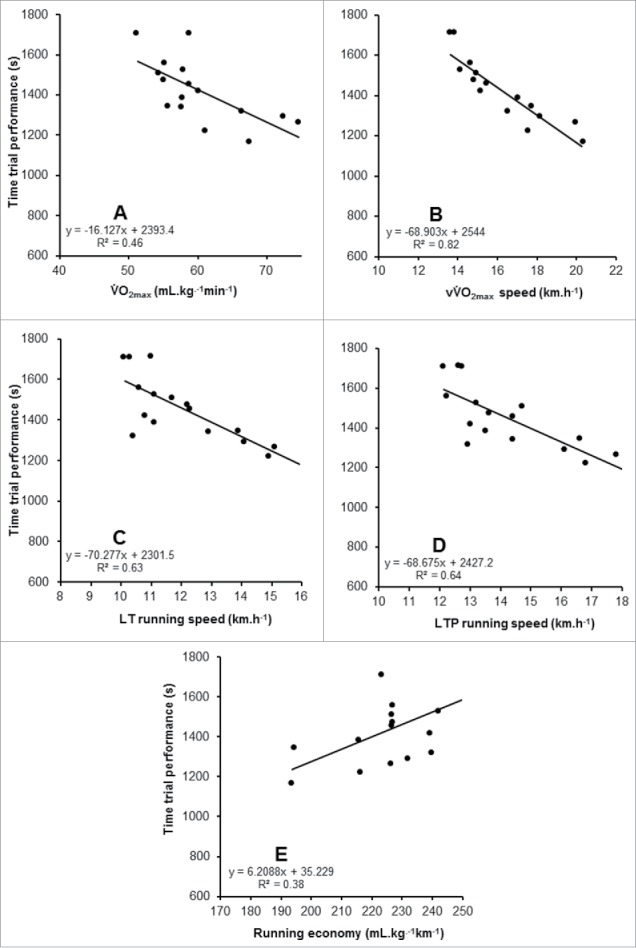
Relationships between the determinants of endurance performance when measured in a cool environment (GXT_COOL_) and 5-km time trial performance in the heat. A – V̇O_2max_, B – vV̇O_2max_, C – lactate threshold (LT), D – lactate turnpoint (LTP), E – running economy.

When measured in GXT_COOL_, LT, LTP, RE, V̇O_2max_, and vV̇O_2max_ were all statistically significant predictors of TT performance in the heat (Table 3 and Fig. 5). Again, the vV̇O_2max_ was the strongest predictor of 5-km performance in GXT_COOL_ (*r* = −0.90). The results from GXT_HOT_ and GXT_COOL_ are shown in Table 3. To compare against the results of GXT_HOT_, that did not include RE in the predictive model, a simultaneous multiple linear regression was completed, excluding RE, revealing a significant relationship with TT performance (F_[4, 10]_ = 11.396, *p* = 0.001, R^2^ = 0.82, SEE = 85 s). Subsequently, adding RE to these variables further improved the model (F_[5, 9]_ = 11.465, *p* = 0.001, R^2^ = 0.86, SEE = 78 s). The model revealed the following formula for predicting 5-km performance based on measures derived from GXT_COOL_.

$$\text{5}\;\text{km}\;\text{time}\;\left(\text{s}\right)=-\text{220}\text{.569}-\text{(31}\text{.288}\;\dot{\text{V}}{\text{O}}_{\text{2max}})\text{+(40}\text{.994}\;\text{v}\dot{\text{V}}{\text{O}}_{\text{2max}}\text{)}-\text{(43}\text{.484}\;\text{LT)}+\text{(61}\text{.911}\;\text{LTP)}+\text{(11}\text{.117}\;\text{RE)}\text{.}$$

## Discussion

This study investigated the relationship between the determinants of endurance performance, when measured in hot and cool conditions, and 5-km running TT performance in hot conditions. In GXT_HOT_, the determinants explained 72% of performance and 82% in GXT_COOL_. These findings indicate that the incremental running test in hot conditions is a weaker predictor of performance in the heat compared with testing in cool conditions. Supporting previous research conducted in cool conditions,^6,8,37,38^ vV̇O_2max_ appears the strongest predictor of 5-km performance in the heat. The overall models derived from both GXT_HOT_ and GXT_COOL_ represent weaker relationships than are previously reported when the GXT and TT occur under conditions without heat stress,^8^ alluding to greater unexplained variance when endurance running performance occurs in the heat.

### Predicting running performance in the heat

In GXT_HOT_, vV̇O_2max_, V̇O_2max_, LT, and LTP explained 72% of the variance in 5-km performance, indicating that the majority of endurance performance in the heat is still underpinned by the traditional physiologic determinants. However, the prediction model reveals greater unexplained variance than previously reported for 16-km running performance (R^2^ = 0.9788) and did not include RE due to the absence of a significant linear relationship for RE to predict 5-km performance. It should be acknowledged there is likely a greater anaerobic contribution during a 5-km TT rather than 16 km,^39^ while the study of McLaughlin et al.^8^ also involved over-ground, rather than treadmill running. However, as shown in Table 3, some relationships between individual predictors and performance are also weaker than previously reported, suggesting that other factors elicit a greater influence on endurance performance in the heat, than as previously reported in cool conditions. Moreover, the SEE of the derived equations is of sufficient magnitude that predictions would not be meaningful, given our laboratory typical error for this trial (16 s, 1.2%).

A stronger model was observed from GXT_COOL_ (GXT_HOT_ R^2^ = 0.72, GXT_COOL_ R^2^ = 0.82), indicating TT performance in the heat is not as closely associated to the physiologic responses during GXT_HOT_, as when the determinants are assessed in GXT_COOL_. One explanation could be the fixed GXT intensities not eliciting analogous decrements on the determinants across participants, as demonstrated in Fig. 3. In compensable heat stress environments, fixed exercise intensities, as adopted in the GXT, can elicit different magnitudes of heat strain between individuals, due to differences in body mass^40^ and/or training status.^41^ This highlights the potential for individual variation in the changes in the determinants of endurance performance, when measured in GXT_HOT_, relative to GXT_COOL_, weakening the prediction model of GXT_HOT_. The differences between GXT_HOT_ and GXT_COOL_ cannot be explained by the addition of RE to the model, because while RE strengthens the prediction of performance, the smaller model derived from V̇O_2max_, vV̇O_2max_, LT, and LTP already explained a greater proportion of variability and demonstrated a lower SEE in GXT_COOL_. There were very high-sample correlations among many of these physiologic variables, in particular the running velocity variables, as has previously been reported when multiple independent variables are derived from the same individual.^42,43^ This makes it difficult to assess the relative contribution of each predictor to the overall model,^42^ however, inference can be taken from individual Pearson corelations between each predictor and 5-km performance as shown in Figs. 4 and 5 for GXT_HOT_ and GXT_COOL_, respectively.

Correlation coefficients between 5-km performance and blood lactate indices LT and LTP are stronger than for V̇O_2max_ or RE, but appear slightly weaker than previous research in normothermic conditions (Table 3). Notably, these relationships are also weaker than Lorenzo et al.,^13^ who demonstrated relationships of *r* = 0.87, 0.86, and 0.86 for the lactate inflection point (increase >0.2 mmol.l^−1^), power output at 1 mmol.l^−1^, and power output at 4 mmol.l^−1^ respectively, to predict cycling performance in hot conditions (38°C, 30% RH). Based upon these data, Lorenzo et al.^13^ indicated lactate thresholds should be assessed in the environmental conditions where performance will occur, to accurately predict performance. However, our data indicates a lessened ability of blood lactate indices to predict performance in shorter, running events that are completed at a greater exercise intensity than the 1-hour cycling TT of Lorenzo et al.^13^ The intensity of 5-km running is thought to represent 94–98% of V̇O_2max_ in elite athletes^39^ and corresponded to a mean of 94% of HR maximum in our cohort. Moreover, running elicits greater heat strain than cycling,^44^ likely due to the greater metabolic heat production and reduced convective cooling, while our protocol was also significantly shorter than Lorenzo et al.,^13^ lasting ∼23 min in the heat. Finally, Lorenzo et al.^13^ pre-warmed individuals for 30 min, which may have resulted in a more uniform elevation of both T_SKIN_ and T_CORE_ than our participants experienced while running during GXT 1.

There is generally considered to be a strong inverse relationship between V̇O_2max_ and TT performance within runners of heterogeneous performance levels (*r* = −0.81–0.91),^8,38,42,45,46^ with a high V̇O_2max_ considered a pre-requisite for elite endurance performance, to meet the estimated energy requirements necessary to sustain running velocities observed in high-level competition.^47^ While the relationship between V̇O_2max_ and performance may be weaker within an elite population due to the relative homogeneity of V̇O_2max_ within this cohort,^37^ Billat et al.^48^ demonstrated that V̇O_2max_ may still explain up to 59% of the variation in performance among elite marathon runners. The relationships between V̇O_2max_ and performance in the heat (GXT_HOT_ *r* = 0.70, GXT_COOL_ *r* = 0.67) are weaker than that for 16-km running in the cool (*r* = −0.91),^8^ but stronger than other studies using cohorts who display both heterogeneous V̇O_2max_ and running performance (*r* = 0.559; *r* = 0.51^49^), which may reflect the significant cardiovascular strain observed during exercise in the heat,^50^ indicating a larger V̇O_2max_ to be desirable for performance in the heat.

Running economy during GXT_HOT_ revealed no relationship with performance (*r* = 0.36), unlike in GXT_COOL_ (*r* = 0.62), indicating RE assessment in hot conditions to be inappropriate for predicting performance in the heat. The influence of heat stress on RE is equivocal, possibly due to the progressive onset of heat strain and associated thermoregulatory responses, which may contribute to different outcomes between studies adopting different methodologies. For example, the relatively low starting intensities during the GXT may initially afford a benefit to RE through a more efficient muscle,^51^ before energy demanding thermoregulatory responses such as hyperthermic hyperventilation,^52^ enhance physiologic strain. Therefore, the measurement of RE during GXT_HOT_ may not replicate the physiologic strain experienced during the TT, where a high intensity is observed from the outset (∼94% maximum HR), eliciting a faster and heightened thermoregulatory response, despite matched heat stress. It is also plausible that GXT_COOL_ elicited a small reduction in starting muscle temperature, affording a small impairment to muscle efficiency and requiring greater oxygen consumption for a given running speed, although we acknowledge muscle temperature was not measured. Notwithstanding, GXT_COOL_ demonstrated a stronger relationship between RE and performance, but this remains a relatively weak relationship, reaffirming RE to be a weak predictor of 5 km or similar distances.^9,49^ This is unsurprising, given that RE is most related to longer distance events than 5 km^39^ and would appear to exert the greatest influence within a group of athletes with relatively homogenous V̇O_2max_,^37^ which was not the case in this sample (V̇O_2max_ range 51–75 mL.kg^−1^min^−1^).

Despite the limitations of the relationship between RE and endurance performance, vV̇O_2max_ remained the strongest predictor, both when derived from GXT_HOT_ and GXT_COOL_, which is in broad agreement with previous literature in Table 3, and the study of Houmard et al.^53^ who reported vV̇O_2max_ to explain 92% of the variance in 8-km performance. This reaffirms the importance of training and monitoring of the parameters that determine vV̇O_2max_ for improving 5-km performance in the heat. However, this observation should not necessarily reduce the importance attributed to blood lactate thresholds, given that Morgan et al.^37^ have previously highlighted the intuitive notion that a direct link may exist between vV̇O_2max_ and determinants of blood lactate indices, such as capillary density, fiber-type distribution, respiratory capacity, and muscle enzyme activity, suggesting that training of either V̇O_2max_, RE, or lactate thresholds may provide mutual benefits.

### Unexplained variance

In comparison to the data of McLaughlin et al.,^8^ who adopted a comparable participant cohort and similarly analyzed the entire model of the traditional determinants of endurance performance, our data from both GXT_HOT_ and GXT_COOL_ explained less of the variance in performance. When exercising under heat stress, it has previously been shown that perceived thermal and/or exertional strain exert marked influences on self-selected exercise intensity, in advance of changes in body temperature, through to moderate levels of hyperthermia.^17,20,54^ Such adjustments to exercise intensity represent a behavioral response, and in the heat likely reflect behavioral attempts to thermoregulate and/or alleviate unfavorable sensations.^16^ Therefore, while acknowledging differences in energetic profile of the event lengths, behavioral thermoregulation, presenting as a reduction in the self-selected running speed, is a likely candidate to contribute to the unexplained variance in determining endurance performance in the heat.

Behavioral thermoregulation has previously been suggested to be a determinant of exercise performance under heat stress.^16^ For example, in high-level athletes, the trend toward a flatter pacing profile following familiarization to endurance exercise in the heat,^55^ represents a form of behavioral thermoregulation as individuals seek to avoid beginning exercise at an intensity that may yield a subsequent disadvantage arising from excessive heat storage. In our 5-km TT, participants may have altered running speed in accordance with perceived thermal discomfort, rather than solely in accordance with the physiologic markers that largely determine performance in cooler environments. The concept of a physiologic reserve when exercising in the heat is supported by the effects reported following the ingestion of dopamine/noradrenaline reuptake inhibitor, that facilitated improved TT performance in the heat, for the same perceptual responses, but not in cool conditions.^56^ Flouris and Schlader^16^ have suggested thermal discomfort may be a contributing factor to RPE, which may be the ultimate moderator of behavioral thermoregulation, to which both physiologic strain and unfavorable sensations feed into. RPE may initially be primarily influenced by T_SKIN_, with T_CORE_ exerting a greater influence as hyperthermia progresses.^16^ During the 5-km TT, as T_CORE_ elevation and hyperthermia became more pronounced, physiologic strain may have influenced performance both directly, through limiting aerobic capacity, but also indirectly by enhancing RPE.^20,57^ Therefore, in the heat, RPE may encompass both physiologic strain, as well as perceived strain, that may occur somewhat independently, during exercise in the heat. However, we cannot infer a relationship between RPE during the GXT and the TT because RPE measured during an incremental exercise test may not replicate prolonged exercise protocols, especially under heat stress^58^ and may be susceptible to bias from prior knowledge of the protocol length.^59,60^ Therefore, associations between RPE or other ordinal level data such as thermal sensation and the response during the TT are not possible.

### Limitations

As a 5-km TT was not completed in cool conditions, the efficacy of determinants of endurance performance for predicating performance for this cohort outside of heat stress is unknown. It is also acknowledged that exercise tests were conducted without representative air flow, which will likely impair convective cooling^61^ and, in turn, may have exacerbated physiologic strain and thermal discomfort, relative to outdoor running. Therefore, future research should replicate these exercise tests under conditions incorporating suitable airflow. Finally, relationships are derived from treadmill running, which although modified to replicate the increased energy expenditure of outdoor running,^27^ and valid and reliable for assessing endurance running performance,^62^ may be insensitive to small, intuitive changes in running speed.^63^ To mitigate this, participants were asked to practice both small and large adjustments in treadmill speed during their familiarization, as well as being reminded they were free to adjust the speed as much, or as little as they liked before every trial.

### Practical applications

Event characteristics such as distance and duration may determine whether it is appropriate to conduct a laboratory test in representative environmental conditions, due to the potential for heat stress to afford a transient improvement to running economy that does not appear to replicate 5-km TT exercise. Therefore, completing TT in the heat may be more appropriate for assessing training status in competitive athletes before competing. When laboratory testing does take place, the best single predictor of 5-km performance would appear to be vV̇O_2max_, measured in cool conditions. The traditional determinants of endurance performance, vV̇O_2max_, V̇O_2max_, LT, and LTP, appear to remain prerequisites, accounting for 82% of variance in performance when measured in GXT_COOL_, emphasizing the importance of continuing to train these areas. However, our study also alludes to prioritizing improved perceived thermal and/or exertional strain, to minimize behavioral attempts to thermoregulate. Therefore, monitoring thermal sensation, comfort and RPE relative to fixed velocities and durations during an athlete's training program in a hot environment (i.e., heat acclimation) would appear useful to track improvement. Furthermore, both short- and long-term acclimation training appear effective strategies for improving perceived thermal strain,^21,64-66^ as may the acute approach of adopting a menthol mouth rinse.^67^ During acclimation training, the adoption of high humidity conditions or using ergogenic aids, such as sauna suits,^68^ that minimize heat loss, may be effective methods of accentuating perceived thermal strain, due to the potential link between thermal comfort and skin wetness.^69^

## Conclusion

In conclusion, predicting running performance in the heat from GXT_COOL_ appears more appropriate than GXT_HOT_, possibly due to the progressive onset of heat strain not replicating that of the time trial. The vV̇O_2max_ also appears to remain the best predictor, when running endurance performance occurs in the heat. Finally, the model of the traditional determinants of endurance running performance; vV̇O_2max_, V̇O_2max_, RE, LT, and LTP, appear pre-requisites for endurance performance in the heat, but may explain less variance in performance than previously reported in cool conditions, albeit over longer distances.

## Abbreviations

GXT: Graded exercise tesp
GXT_COOL_: Graded exercise test conducted in cool environment
GXT_HOT_: Graded exercise test conducted in hot environment
HR: Heart rate
LT: Lactate threshold
LTP: Lactate turnpoint
RE: Running economy
RER: Respiratory exchange ratio
RH: Relative humidity
RPE: Rating of perceived exertion
T_CORE_: Core temperature
TS: Thermal sensation
T_SKIN_: Skin temperature
TT: Time trial exercise protocol
V̇O_2_: Oxygen consumption
V̇O_2max_: Maximal oxygen consumption
V̇O_2peak_: Peak oxygen uptake
vV̇O_2max_: Estimated velocity at maximal oxygen consumption
WBGT: Wet bulb globe index
